# Anesthetic Management of a Pediatric Patient With Mitochondrial Depletion Syndrome and Hypertrophic Cardiomyopathy Undergoing Scoliosis Correction: A Case Report

**DOI:** 10.7759/cureus.88669

**Published:** 2025-07-24

**Authors:** Ali Alkuwaiti, Muneera Albuthi, Ahmed Haroun M Mahmoud, Ghidaa Gosty, Abdulrahman A Alselaiti

**Affiliations:** 1 Pediatric Anesthesia, King Abdulaziz Medical City Riyadh, Riyadh, SAU; 2 Medical Education, College of Medicine, King Abdulaziz Medical City Riyadh, Riyadh, SAU; 3 Research and Development, King Abdullah International Medical Research Center, Riyadh, SAU; 4 Pediatric Anesthesiology, King Abdullah Specialized Children's Hospital, Riyadh, SAU; 5 College of Medicine, King Saud Bin Abdulaziz University for Health Sciences, Riyadh, SAU; 6 Anesthesia, King Abdulaziz Medical City Riyadh, Riyadh, SAU

**Keywords:** anesthesia, mitochondrial depletion, pediatric, scoliosis correction, syndrome

## Abstract

This report describes a case of a 15-year-old pediatric patient with mitochondrial depletion syndrome, associated with hypertrophic cardiomyopathy, who underwent scoliosis correction surgery under neurophysiological monitoring. As propofol is mitochondrion-toxic, the anesthetic approach was modified to involve dexmedetomidine infusion combined with boluses of midazolam and ketamine. The patient was hemodynamically stable during surgery with effective neuromonitoring readings, and the surgery was concluded uneventfully. This case highlights the safety and efficacy of this anesthetic regimen in managing patients with complex metabolic and cardiac conditions.

## Introduction

Mitochondrial depletion syndrome is a rare disorder characterized by impaired energy production due to mitochondrial dysfunction that often complicates anesthetic management. The mitochondrial production of adenosine triphosphate (ATP) is the primary source of energy for intracellular metabolism. Precisely, the human mitochondrial ATP synthase, also known as complex V, is the fifth multi-subunit complex involved in oxidative phosphorylation (OXPHOS) [1,2]. Mitochondrial diseases are classified into three primary categories: mitochondrial respiratory chain disorders, mitochondrial deletions and depletions, and mitochondrial membrane disorders [3]. Mitochondrial anomalies associated with energy metabolism deficiencies increase the risk of several disorders involving multiple body systems, like hypertrophic cardiomyopathy. Mitochondrial disorders impose significant risks and concerns for anesthesiologists due to the heightened metabolic stress and associated comorbidities during surgical interventions [4].

This report describes a high-risk pediatric patient with mitochondrial depletion syndrome who underwent a posterior spinal fusion corrective surgery with the successful use of dexmedetomidine, midazolam, and ketamine during intraoperative management that required neuromonitoring, emphasizing the importance of tailored anesthetic strategies.

## Case presentation

**Figure 1:**
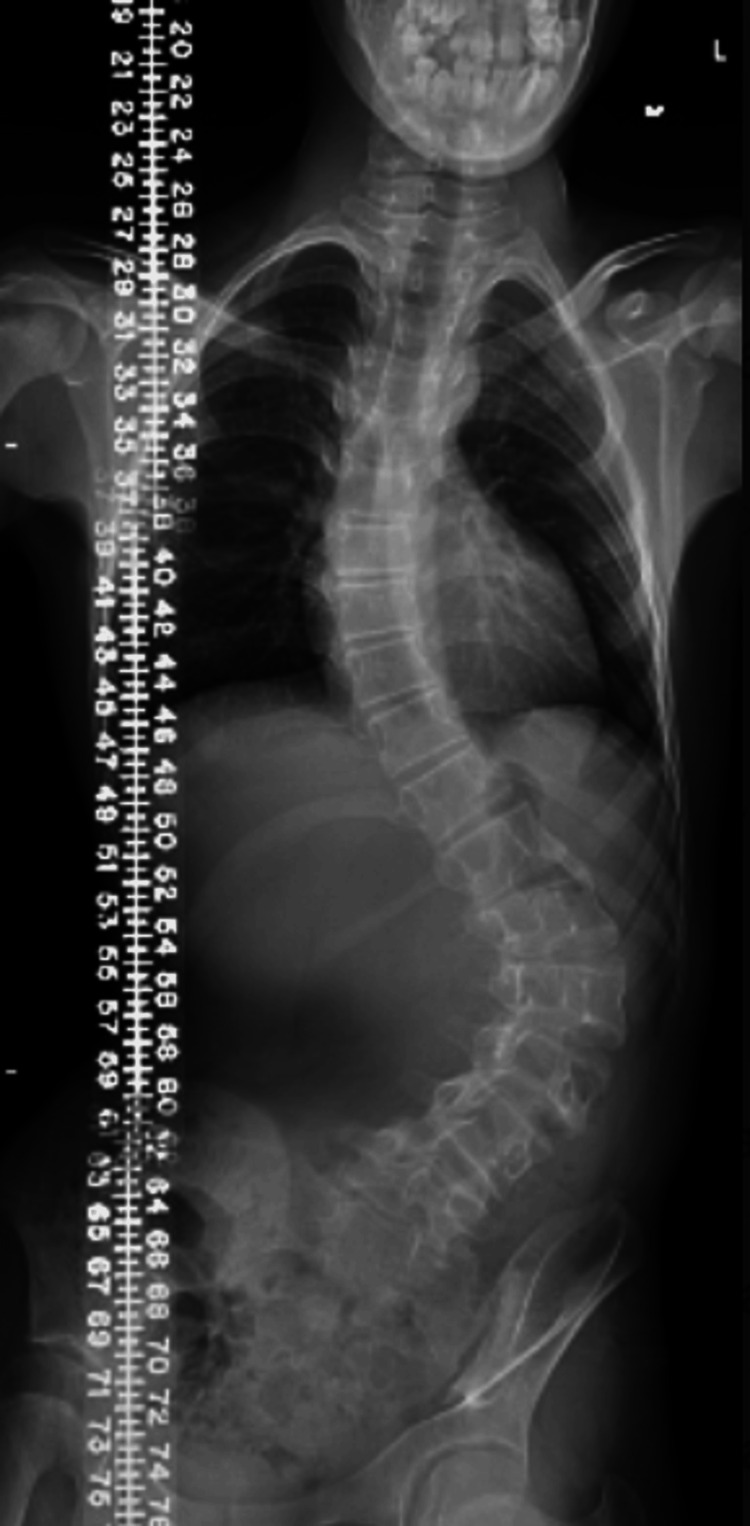
Radiograph of the thoracolumbar spine with S-shaped scoliosis.

**Figure 2:**
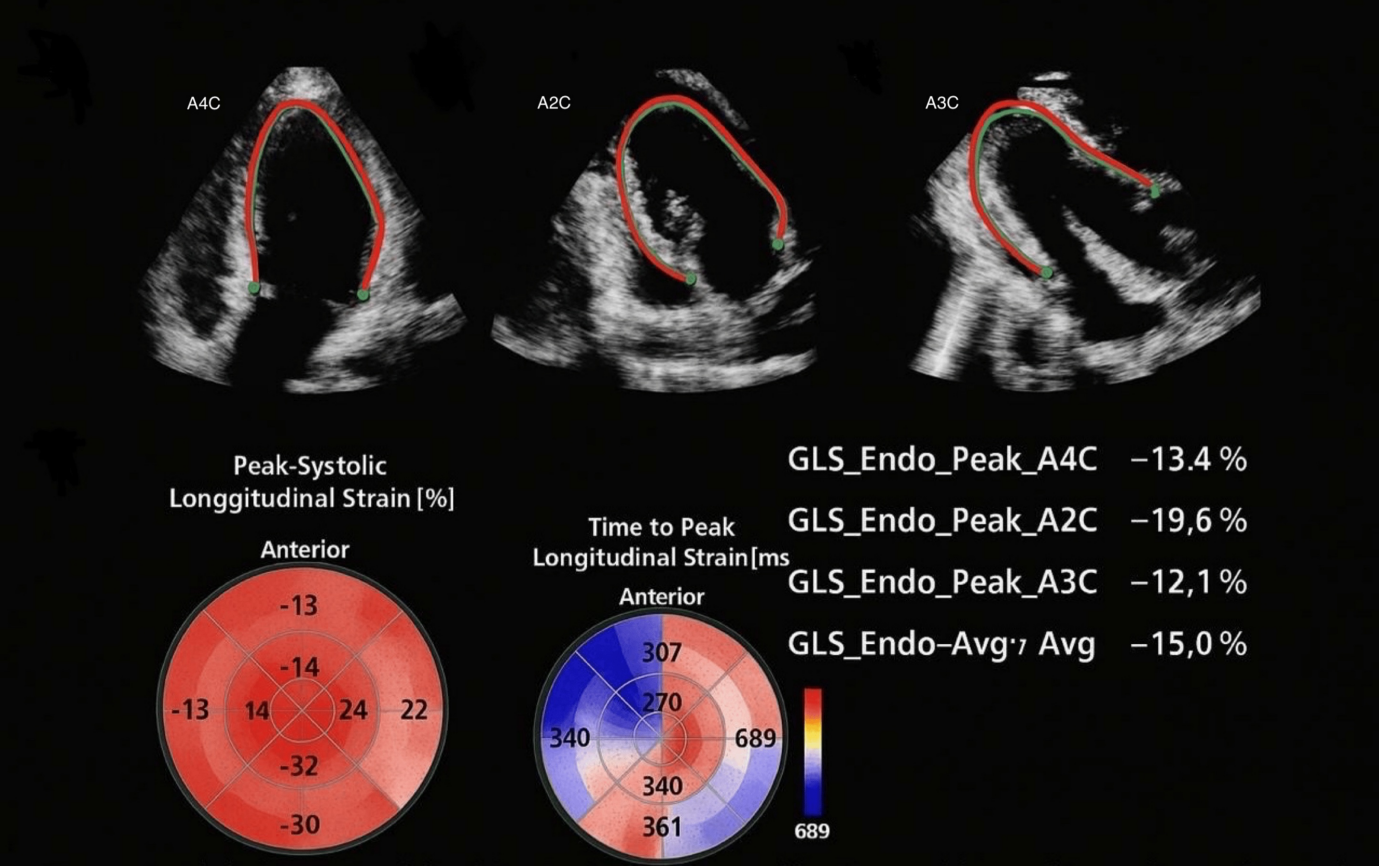
Global longitudinal strain (GLS) assessed by 2D tracking echocardiography. A4C: apical four-chamber view; A2C: apical two-chamber view; A3C: apical three-chamber view.

**Figure 3:**
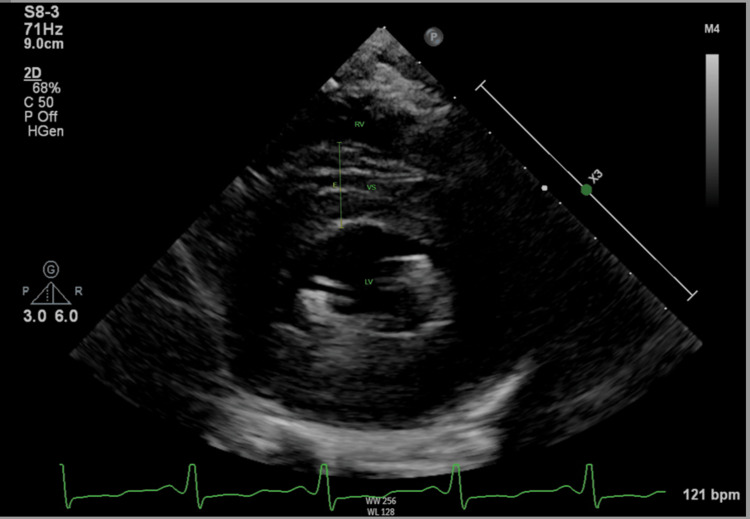
Echocardiography short-axis view showed septal hypertrophy at mid-basal, measuring 17.2 mm, during the systolic phase. RV: right ventricular; VS: ventricular septum; LV: left ventricular.

A 15-year-old male with mitochondrial depletion syndrome complicated by hypertrophic cardiomyopathy presented for corrective surgery of thoracolumbar levoscoliosis (Figures 1-3). He was diagnosed with mitochondrial depletion syndrome, specifically mitochondrial complex V deficiency, at the age of three years. Further cardiac evaluation revealed hypertrophic cardiomyopathy with limited, yet stable functional capacity. Additionally, the patient’s past medical history was found to include cerebral palsy, intellectual disabilities, and attention-deficit hyperactivity syndrome. Surgically, he had no previous history.

Regarding his medications upon admission, he was on carnitine, thiamine, and riboflavin. Preoperative assessments had been done and cleared by the genetic and cardiology departments for the elective procedure of posterior spinal fusion at the T2-S2 levels. From a laboratory perspective, complete blood count, metabolic panel, liver function tests, and coagulation panel tests were within normal limits with normal lactate levels and mildly elevated creatine kinase at 300 U/L (normal: 30-200 U/L). Moreover, echocardiography was confirmatory for left ventricular hypertrophy without outflow obstruction with left ventricular ejection fraction (EF) of 53%. The booking of the procedure was completed with the reservation of a pediatric intensive care unit (PICU) bed for postoperative care and blood product allocations. The preanesthesia plan was conducted to use fentanyl, lidocaine, ketamine, midazolam, and rocuronium for induction. During maintenance, total intravenous anesthesia (TIVA) of dexmedetomidine and fentanyl infusions combined with boluses of midazolam and ketamine were used to facilitate the neurophysiological monitoring.

Preoperatively, the patient’s vital signs were stable, with blood pressure at 115/55 mmHg, heart rate of 112 bpm, respiratory rate of 20 bpm, oxygen saturation at 97% on ambient air, and temperature at 36.9°C. The patient’s weight recorded was 30 kg. In the holding area, 1 mg of midazolam was administered. Upon entrance to the operation theater, the American Society of Anesthesiologists (ASA) standard means of monitoring, including continuous electrocardiogram (ECG) monitoring, non-invasive blood pressure (NIBP) measurement every five minutes, continuous pulse oximetry for oxygenation, continuous capnography for ventilation assessment, and temperature monitoring, were applied in addition to the bispectral index (BIS) monitor.

Intraoperatively, general anesthesia was induced using ketamine (1 mg/kg) and midazolam (0.05 mg/kg), supplemented with 60 µg of fentanyl, 30 mg of lidocaine, and 10 mg of rocuronium. Intubation was performed via direct laryngoscopy using a size 7 reinforced endotracheal tube. Following induction, a 22-gauge left radial artery line, a 22-gauge peripheral intravenous line, and a 5 Fr × 13 triple-lumen internal jugular central catheter were inserted. Anesthesia was maintained with TIVA infusion of dexmedetomidine at 0.7 µg/kg/hr and fentanyl at 3-5 µg/kg/hr, titrated as required, along with boluses of midazolam (0.05 mg/kg/hr) and intermittent ketamine boluses (0.3 mg/kg/hr) for analgesia. Fluid management included a 5% dextrose infusion in normal saline at 70 mL/hr. For neurophysiological monitoring, the motor evoked potentials (MEPs) and somatosensory evoked potentials (SSEPs) were assessed and showed no impairments, remaining stable throughout the procedure. The electroencephalography (EEG) showed no epileptiform activity. Neurophysiological monitoring remained undisturbed with no adverse effects from midazolam, ketamine, fentanyl, or dexmedetomidine. Hemodynamically, the patient remained stable with a mean arterial pressure sustained above 60 mmHg and a heart rate of 110 bpm, without requiring vasoactive support. As shown in Table 1, serial arterial blood gas readings were within the normal range throughout the case. For instance, the baseline lactate level was 1.5, peaked at 1.7, then decreased to 1.1 by the end of the case. The procedure took around seven hours; both dexmedetomidine and fentanyl infusions were stopped one hour before the end of the procedure. Blood loss of 200 mL was reported, and fluid maintenance and deficit were replaced by the total amount of 5% human albumin 300 mL (10 mL/kg) and PlasmaLyte 1470 mL (7 mL/kg/hr), in addition to 70 mL/hr 5% dextrose infusion in normal saline. Postoperatively, the patient was extubated in the operating theater and subsequently transferred to the PICU for observation. After a stable two-day stay in the PICU, the patient was moved to the general pediatric ward, where recovery continued uneventfully over the following five days. The patient was then discharged home in stable condition with no signs of lactic acidosis or cardiac decompensation.

**Table 1:** Intraoperative arterial blood gas (ABG) values. PaCO_2_: partial pressure of carbon dioxide; PaO_2_: partial pressure of oxygen; HCO_3_: bicarbonate; SatO_2_: oxygen saturation; Hgb: hemoglobin; O2Hb: oxyhemoglobin; MetHb: methemoglobin; COHb: carboxyhemoglobin.

Parameters	Starting	Middle	End	Reference values
pH	7.45	7.36	7.4	7.35-7.45
PaCO_2 _(mmHg)	33	41	35	32.0-48.0
PaO_2 _(mmHg)	204	191	150	83.0-108.0
HCO_3_-	22.9	23.3	21.7	22-26
SatO_2 _(%)	99.6	99.9	99.3	95-100
Hgb (g/dL)	10.8	9.8	8.2	11.7-15
O2Hb	98.1	97.9	97.9	95-100
MetHb	0.9	0.9	0.9	0-1.5
COHb	0.7	1.2	0.8	0.5-1.5
Lactate (mmol/L)	1.5	1.7	1.1	0.20-1.80

## Discussion

This case report highlights the use of dexmedetomidine as the primary sedative in a high-risk patient, offering hemodynamic stability and minimal interference with neurophysiological monitoring.

TIVA using dexmedetomidine and ketamine was used as an alternative to propofol in a patient with mitochondrial depletion syndrome, a condition associated with significant risks of adverse effects and complications. Mitochondrial disorders are particularly susceptible to anesthesia and surgical stress due to the mitochondria's critical role in supplying energy to high-demand organs like the brain and heart. In such cases, the failure to produce sufficient energy exacerbates metabolic stress intraoperatively and disrupts key biochemical processes. For example, a compromised oxidative phosphorylation process leads to increased lactate production, thereby exacerbating lactic acidosis [5].

Multiple reports have demonstrated that propofol infusion in patients with mitochondrial disorders has impaired mitochondrial activity and increased susceptibility to propofol infusion syndrome, a life-threatening condition of serious lactic acidosis and cardiac failure [6,7]. However, one study showed that small intravenous boluses of propofol, in addition to ketamine and benzodiazepines, were safe to use and associated with no recognizable decompensation [8]. In this case, ketamine provided effective analgesia while avoiding respiratory depression or mitochondrial dysfunction exacerbation [6].

Prior to the operation, it is important to optimize the patient’s condition through comprehensive care strategies that decrease the metabolic burden. A multidisciplinary approach involving the patient-physician, anesthesiologist, cardiologist, neurologist, endocrinologist, and geneticist should collaborate to devise the safest approach for the procedure and communicate the potential benefits and risks to the parents. Preoperative investigations, including complete blood count, chemistry, blood gas analysis, electrocardiogram, echocardiography, and spirometry, should be conducted. Early patient admission of at least one day before the surgery is advised to allow re-evaluation. Means to decrease the risks of metabolic burden include avoiding hypoglycemia, hypothermia, acidosis, and hypovolemia [6]. Hypoglycemia may be avoided by scheduling the patient first in the operation list, administering glucose-containing clear oral fluid two hours prior to the procedure, and providing appropriate maintenance glucose infusion during the operation. Normothermia can be accomplished by continuous temperature monitoring and management. For fluid maintenance, lactic acidosis can be avoided by the replacement of Ringer’s lactate with PlasmaLyte and albumin. Also, respiratory acidosis can be controlled by adjusting the mechanical ventilation to maintain normocarbia [3]. Hypovolemia may be prevented by sustaining a mean arterial pressure above 60 mmHg. It is critical to have a series of arterial blood gas analyses to monitor lactate levels and identify pH changes, as this ensures prompt and adequate metabolic management throughout the procedure.

Furthermore, intraoperative neuromonitoring is of invaluable use during spine procedures to ensure appropriate motor and somatosensory function and maintain the patient’s safety [9]. While propofol is commonly used to achieve adequate signal attainment, dexmedetomidine and fentanyl showed none to minimal interference with neurophysiological monitoring [10]. While volatile anesthetic agents have not been shown to be harmful for patients with mitochondrial disorders, their interference with neuromonitoring led to their avoidance in this case [10].

## Conclusions

In conclusion, this case underscores the importance of individualized anesthetic management in patients with mitochondrial disorders undergoing surgical procedures. The combination of dexmedetomidine, midazolam, and ketamine proved to be a safe and effective anesthetic strategy for pediatric patients with mitochondrial depletion syndrome and hypertrophic cardiomyopathy undergoing scoliosis correction. Meticulous monitoring of metabolic parameters further contributed to favorable outcomes. This approach ensured hemodynamic stability, preserved neuromonitoring integrity, and minimized the risk of metabolic complications, reinforcing its suitability for this high-risk population. Further research is recommended to refine and optimize anesthetic strategies for this vulnerable population, enhancing safety and improving perioperative outcomes.
